# The Role of Cost-Effectiveness Analysis in Patient-Centered Cancer Care in the Era of Precision Medicine

**DOI:** 10.3390/cancers13174272

**Published:** 2021-08-25

**Authors:** Fabrizio Toscano, Alberto Vera, Eleanor Kim, Davide Golinelli, Helena Vila-Reyes, Fernand Bteich, Antoine Schernberg, Romain-David Seban, Randy Yeh, Laurent Dercle

**Affiliations:** 1 Division of General Internal Medicine, Montefiore Medical Center, Bronx, NY 10467, USA; albertovera19@gmail.com; 2 Department of Radiology, New York Presbyterian, Columbia University Irving Medical Center, New York, NY 10032, USA; ehk7001@nyp.org (E.K.); hv2208@cumc.columbia.edu (H.V.-R.); 3 Department of Biomedical and Neuromotor Sciences, University of Bologna, 40126 Bologna, Italy; davide.golinelli@unibo.it; 4 Department of Oncology, Montefiore Medical Center, Bronx, NY 10467, USA; fbteich@montefiore.org; 5 Department of Radiation Oncology, Centre de Cancérologie de la Porte de Saint-Cloud, 92100 Boulogne-Billancourt, France; aschernberg@gmail.com; 6 Department of Nuclear Medicine and Endocrine Oncology, Institut Curie, 92210 Saint-Cloud, France; romaindavid.seban@curie.fr; 7 Laboratoire d’Imagerie Translationnelle en Oncologie, Inserm, Institut Curie, 91400 Orsay, France; 8 Department of Radiology, Memorial Sloan Kettering Cancer Center, New York, NY 10065, USA; yehr@mskcc.org

## 1. Introduction

Over the last few decades, changes in diagnostic and treatment paradigms have greatly advanced cancer care and improved outcomes [1]. However, these advances have dramatically increased cancer care costs for patients and society. The United States (US) National Cancer Institute estimates that cancer care costs will rise to USD 246 billion by 2030, a 34% increase from a 2015 figure of USD 183 billion [2], perceived by some as conservative in the context of the alarming pace at which cancer-attributable medical costs have been increasing.

**Figure 1:**
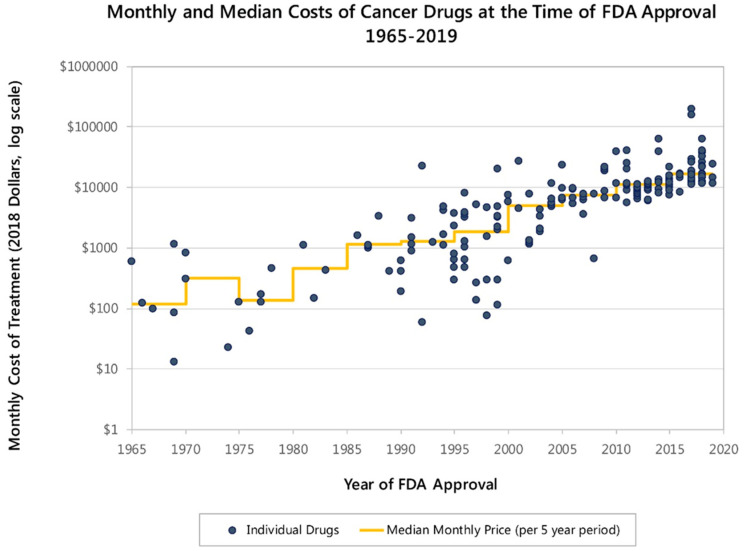
Monthly and median costs of cancer drugs at the time of FDA approval (1965–2019). Courtesy of Peter B. Batch, MD, Memorial Sloan Kettering Cancer Center. Source: https://www.mskcc.org/research-programs/health-policy-outcomes/cost-drugs (accessed on 14 July 2021).

To best illustrate this problem, immunotherapy, arguably the biggest advent in cancer pharmacological research within the past two decades, has been regularly pointed to as a possible cause for this sharp rise in cancer care costs (Figure 1). The technological progress involved in developing these drugs, paired with the relative affordability of genomic analysis, made possible the concept and promise of precision medicine in cancer care, increasing patient and physician expectations in parallel [3].

With more and more immunotherapeutic options available and with the average cost of these solutions being USD 145,000 per patient per year [4], pharmaco-economic evaluations seem to be an obvious necessity. The goal of these analyses is to determine if one treatment offers the highest return in health gain for its cost. Such cost-effectiveness analyses are often used by health technology assessment agencies, but the consequences of their findings vary from one country to another. In addition to immunotherapeutic agents, recent advances in radiation therapy have led to a substantial increase in cancer care costs as well [5]. Indeed, novel technologies such as stereotactic body radiation therapy, intensity-modulated radiation therapy, volumetric-modulated arc therapy, or stereotactic radiosurgery are more effective and/or less toxic than the historical 3-dimensional conformal radiotherapy [6]. In localized prostate cancer patients, these technical advances have been demonstrated to be cost-effective [7], but this does not always hold true. For instance, proton therapy boomed in the 2000s, particularly in prostate cancer radiation therapy, but the cost-effectiveness ratio was subsequently shown to be unfavorable [8]. However, for certain tumor localizations such as with head and neck cancers, proton therapy could prove to have a favorable cost-effectiveness ratio, which would make this an option of interest [9].

In the field of surgery, technological advances have also sparked concerns. For instance, in specialties such as Urology, the use of robotic platforms to perform surgeries that could otherwise be carried out in an open/laparoscopic fashion represents a significant financial burden on final expenses [10,11]. The latest “da Vinci” robot model (Intuitive Surgical Inc., Sunnyvale, CA, USA) priced at USD 2 million per platform, with additional expenses on life-limited instruments, maintenance, and specific training contracts, largely contributes to the rising cost per procedure [12]. In a meta-analysis comparing the oncologic outcomes of robotic-assisted and laparoscopic versus open radical prostatectomy, evidence of significant superiority could not be found for any approach [13]. Despite the existing evidence questioning the absolute clinical benefit of robot-assisted approach, its use in prostate cancer surgery has been increasing exponentially in most developed countries since its introduction in 2001. From the surgeon’s perspective, the significantly shorter learning curve, improved vision, and ergonomics are notable when compared to open or laparoscopic surgery, while the positive surgical outcomes (smaller incisions, less blood loss, and shorter recovery time) have been praised by both surgeons and patients when compared to open surgery. What remains a major concern is that it has been replacing more affordable treatments without any significant benefit being proven in the long term [14].

Given the conditions above, along with the fact that cancer is now the second leading cause of death in the US and most developed countries with an increasing number of cancer patients every day, addressing cancer care cost-effectiveness is of paramount importance from the perspective of all parties involved. [15,16].

## 2. Patient Perspective

A month of cancer treatment in the US can cost up to USD 60,000 (which includes not only the cost of medications but also of direct care, consults, imaging, ancillary services, and so on) [17]. Average out-of-pocket expenses for an individual are USD 1,107 for the first year after diagnosis and USD 747 annually thereafter. Some patients must pay up to USD 10,000 in out-of-pocket expenses before comprehensive insurance coverage begins (a sum known as a “deductible”), while other patients reach their annual out-of-pocket maximum in just the first month of treatment. The consequences are devastating: receiving cancer care alone makes a patient more than twice as likely to declare bankruptcy [18]. These patients tend to have a lower quality of life, diminished physical health, and significant psychological distress [19]. Financial toxicity has also been associated with worse outcomes, as affected patients experience a three-fold increase in mortality rates due to overall poorer well-being, impaired health-related quality of life, and sub-par quality of care [20].

Therefore, cost is a critical aspect to consider when delivering cancer care. Interestingly, while most cancer patients (52%) want to discuss costs with oncologists, only a minority (19%) will ultimately have such conversations [21,22]. Identifying barriers to open communication and adopting solutions to address these obstacles may allow for improved clinician–patient shared decision-making and could result in effective clinical outcomes, while reducing the unnecessary financial burden and psychological distress which frequently come from patient uncertainty.

As such, new models for the delivery of cancer care have been explored recently, stemming from the experience of managing other chronic disease patients. The National Committee for Quality Assurance has proposed a list of goals for patient-centered oncology care that aims to address the most common issues that lead to poor outcomes [23], including enhancing access and continuity of care, using data for population management, providing care management, supporting self-care processes, coordinating referral tracking and follow-ups, and implementing continuous quality improvement. While these models for the delivery of cancer care have been showing promising results in increasing the overall quality of care, their financial cost to the patient should also be evaluated [24,25].

## 3. Healthcare Provider Perspective

A national survey conducted in the US in 2008 found that a vast majority of oncologists (84%) made their treatment recommendations based upon patients’ out-of-pocket spending. Strikingly, only a minority (43%) discussed costs with patients [26]. Most providers who responded to the survey based their treatment choice upon comparative effectiveness research (79%) and cost-effectiveness data (80%), yet only a minority felt that they were able to correctly interpret the results (42%). This underscores the critical need for rigorous comparative and cost-effectiveness studies to enhance physician understanding of treatment costs and promote shared decision-making as a means to reduce costs—one of the goals prioritized by the Institute of Medicine to increase the quality of cancer care [20,27].

Cancer patients often see multiple providers and frequently face a myriad of treatment options. Fragmentation of care is an unfortunate result of the current payment structure, which creates an environment that does not allow oncologists—who would otherwise naturally coordinate patient care—to bill for shared decisions, help patients navigate the system, or support them emotionally [25]. These issues of utilization and integration of care are what patient-centered models have been aiming to address.

## 4. Policymaker Perspective in the US and Abroad

According to the National Cancer Institute, the medical cost of cancer care in the US was USD 150.8 billion in 2018 and has been increasing substantially every year, even beyond previous predictions [28]. This fast rise in costs has predominantly been driven by drug and insurance prices that have skyrocketed over the last decade [17].

Since the early 1990s, in order to offer more options to patients and clinicians in a timely manner, the US Food and Drug Administration (FDA) has been increasingly approving oncological drugs through accelerated programs [29]. The rationale behind these approval pathways comes from a better understanding of cancer biology, making phase II trials and their surrogate endpoints sufficient to grant new therapies access to the market [30]. In a recent study, 92 cancer therapies approved for 100 indications between 2000 and 2016 were reviewed [31]. Almost half of the indications (42), mainly hematological therapies, had been approved through an accelerated pathway. Interestingly, the studies evaluated for the approval of these drugs were more commonly single-arm and phase II trials with relatively small sample sizes, while drugs approved for solid tumors more frequently entered the market thanks to randomized controlled trials with larger sample sizes. Several studies have highlighted the importance of having proper post-approval trials to test those accelerated pathway cancer drugs on more comprehensive endpoints such as overall survival [31,32,33].

When it comes to radiation therapy, the treatment cost is highly variable between countries, and within the same country at different hospitals [34,35]. Price transparency could promote both value and effectiveness-based decision-making, while reducing the financial burden of radiation therapy treatments. One way to reduce the cost per treatment is to switch from conventional treatments to hypo-fractionated radiotherapy regimens [34,36]. Although it has been shown that hypo-fractionated irradiation can decrease cost to the patient by up to 40%, healthcare providers actually sustain a financial loss via fee-for-service remuneration, dis-incentivizing this otherwise cost-saving treatment [36].

In the US, there is a critical need to control costs to ensure the sustainability of the healthcare system by reforming the payment structure on a national level. Controlling expenditure is a multifaceted challenge involving a wide range of factors. For example, healthcare spending has mostly increased due to higher drug costs, but there are several other contributing factors such as failure of screening programs, increased cancer prevalence, and increased staff costs, legal costs, and facility fees.

The US healthcare system is currently being stretched thin by the increasing cancer incidence rates and the booming cost of medications and technologies. Moreover, the excessive utilization of inpatient and emergency services resulting from fragmented care delivery are all contributing to the overall financial burden of cancer care. New cancer therapies have taken center stage in the health policy debate of several high-income countries, not just in the US. The approaches chosen to make these agents available to patients vary greatly: what is easily approved with little questioning in one country may be rejected on the grounds of excessive cost in another, until a middle-ground settlement is reached between the authorities and pharmaceutical companies. A critical point to consider in this issue is that pharmaco-economic analyses have to be tailored to each country, factoring in its unique system and patient characteristics. Although this could be an obstacle to implementing global solutions, the principles applied in one country may be transferrable elsewhere.

Across the European Union (EU) and the United Kingdom (UK), the cost of cancer therapy falls on third-party payers, usually either the government or sickness funds. The EU relies on the work of the European Medicines Agency (EMA) for the evaluation and supervision of drugs. Rather than replacing national drug authorities [37], the EMA provides a platform that facilitates and accelerates the approval process of medications within all EU member states. In past years, the EMA received criticism regarding the evidence upon which new cancer treatments had been approved: a study conducted on cancer drugs approved between 2009 and 2013 showed that the majority entered the market without benefit to survival or quality of life [38]. Cost-effectiveness evaluation is not routinely part of the EMA drug approval process, but according to their website, the EMA has been working closely with Health Technology Assessment bodies (HTABs) [39], especially in those member states that have such agencies in place.

A well-known example is the National Institute for Health and Care Excellence (NICE). This UK institution assesses new health technologies and the clinical effectiveness of different interventions. According to a study that compared the EMA’s decisions with those of a state member’s HTABs, overall agreement was found; still, the EMA did not carry out any pharmaco-economic assessments [40]. However, a more recent study highlighted discrepancies in the decisions reached by the EMA and the NICE when focusing on treatments that benefited from accelerated approval pathways to facilitate earlier market access (i.e., “conditional marketing authorization” or “approval under exceptional circumstances”) [41]. These pathways are widely used for oncologic treatments in the EU as well. Even though there is only partial or incomplete data available at the moment of approval, the EMA and the HTABs have agreed to promptly revise their decisions as more data become available in consideration of the time constraint cancer patients are under [42,43]. Nevertheless, there seems to be more scrutiny in the past few months of these accelerated pathways, as depicted by the FDA’s recent broad re-evaluation of approvals for immune checkpoint inhibitors in the treatment of multiple malignancies [44].

Initiated by the European Society for Radiotherapy and Oncology (ESTRO) in 2011, the Health Economics in Radiation Oncology (HERO) project aimed to develop a comprehensive knowledge base and model for economic evaluations of radiation therapy treatments in Europe [45]. The group compared a wide variety of reimbursement systems for radiation therapy, including 41 reimbursement systems within 25 countries [46]. They showed that annual expenditure for radiation therapy in European countries represents on average 7% of their total budget for cancer care. This approach in other areas of cancer care could decrease overall costs significantly, but thus far, similar initiatives of this type do not exist.

Additionally, given the recent political changes that caused the UK to leave the EU, it will be interesting to see how the UK will differ in its approach from the EMA. Moreover, with the rise of costly immunotherapies and the spiraling pressure their cost is applying on GDP allocations for health expenditures, more conversations will definitely need to take place around cost-effectiveness and the threat cancer care costs pose on European healthcare systems.

## 5. Conclusions

In this Special Issue, we look for innovative comparative and cost-effectiveness analysis studies that can guide cancer management decisions and inform health policy. Several management strategies achieve similar performances in terms of disease control and survival for a wide range of indications, and the clinical decision is ultimately based upon the subjective criteria employed by the treating physician. Therefore, this topic aims to share new frameworks and concepts in order to determine the most cost-effective diagnostic and treatment strategies for maximizing the care of cancer patients and the utilization of resources.

The rising costs in oncology have had different effects on the major stakeholders in the healthcare system. From a patient’s perspective, there is a critical need to find solutions that relieve the economic burden and financial toxicity of cancer care as it stands now. It is important to help alleviate the psychological impact of these problems in patients who are concurrently experiencing the anxiety of illness and death. For healthcare providers, there is a need to define cost-effectiveness tools and compare diagnostic and therapeutic strategies in order to improve patient-centered care. On a public health level, allocating resources appropriately and determining the effectiveness of different cancer care options are priorities for ensuring sustainable healthcare systems.

Hence, for all the reasons listed above and many more, there is a critical need to implement cost-effectiveness analyses in order to better inform healthcare decisions in clinical practice. There is a sense of urgency to act sooner rather than later with sustainable solutions, or else we may soon face the crumbling of our healthcare systems.
